# A systematic review: is *Anopheles vagus* a species complex?

**DOI:** 10.1186/s12936-024-04888-0

**Published:** 2024-03-27

**Authors:** Dalilah Dalilah, Din Syafruddin, Irsan Saleh, Ahmad Ghiffari, Leo Vernadesly, Lepa Syahrani, Irdayanti Irdayanti, Chairil Anwar

**Affiliations:** 1 Science and Biomedical Doctoral Programme, Faculty of Medicine, Universitas Sriwijaya, Palembang, Indonesia; 2 Department of Parasitology, Faculty of Medicine, Universitas Sriwijaya, Palembang, Indonesia; 3 Department of Parasitology, Faculty of Medicine, Hasanuddin University, Makassar, Indonesia; 4 Hasanuddin University Medical Research Center (HUMRC), Makassar, Indonesia; 5 Department of Pharmacology, Faculty of Medicine, Universitas Sriwijaya, Palembang, Indonesia; 6 Department of Parasitology, Faculty of Medicine, Universitas Muhammadiyah Palembang, Palembang, Indonesia; 7 Medical Student, Faculty of Medicine, Universitas Sriwijaya, Palembang, Indonesia; 8 Doctoral Program, Department of Biology, Faculty of Mathematics and Natural Science, University of Indonesia, Depok, Indonesia; 9 Eijkman Research Center for Molecular Biology, National Research and Innovation Agency (BRIN), Cibinong, Indonesia

**Keywords:** *Anopheles vagus*, *Anopheles vagus limosus*, *Anopheles limosus*, Species complex, Sibling species, Phylogeny

## Abstract

**Background:**

*Anopheles vagus* (subgenus *Cellia*) has been identified as a vector for malaria, filariasis, and Japanese encephalitis in Asia. Sporozoites of *Plasmodium falciparum* and *Plasmodium vivax* have been found in this zoophilic mosquito in Asia and Indonesia. This study systematically reviews publications regarding *An. vagus* species, variation, bio-ecology, and malaria transmission in various localities in Asia, especially Indonesia, to determine whether the current data support *An. vagus* as a species complex.

**Methods:**

The databases Pubmed, Scopus, Europe PMC, and Proquest were searched to identify information regarding the morphology, karyotypes, polytene chromosome, cross-mating, ecology, and molecular identification of *An. vagus* was then evaluated to determine whether there were possible species complexes.

**Results:**

Of the 1326 articles identified, 15 studies were considered for synthesis. The *Anopheles *spp. samples for this study came from Asia. Eleven studies used morphology to identify *An. vagus*, with singular studies using each of karyotype identification, chromosomal polytene identification, and cross-breeding experiments. Ten studies used molecular techniques to identify *Anopheles* spp., including *An. vagus*. Most studies discovered morphological variations of *An. vagus* either in the same or different areas and ecological settings. In this review, the members of *An. vagus *sensu lato grouped based on morphology (*An. vagus*, *An. vagus vagus*, *An. vagus limosus*, and *An. limosus*), karyotyping (form A and B), and molecular (*An. vagus* genotype A and B, *An. vagus* AN4 and AN5). Genetic analysis revealed a high conservation of the ITS2 fragment among members except for the *An. vagus* genotype B, which was, in fact, *Anopheles sundaicus.* This review also identified that *An. vagus limosus* and *An. vagus vagus* were nearly identical to the ITS2 sequence.

**Conclusion:**

Literature review studies revealed that *An. vagus* is conspecific despite the distinct morphological characteristic of *An. vagus* and *An. limosus.* Further information using another barcoding tool, such as mitochondrial COI and ND6 and experimental cross-mating between the *An. vagus* and *An. limosus* may provide additional evidence for the status of *An. vagus* as a species complex.

## Background

Malaria is an infectious disease caused by *Plasmodium* spp. transmitted by female *Anopheles* mosquitoes. Southeast Asia has the second highest malaria incidence after Africa, with an estimated eight million cases and 11,600 malaria deaths [1]. Globally, a total of 465 *Anopheles* malaria vectors have been identified morphologically, with 70 species from the four subgenera *Anopheles*, *Cellia*, *Kerteszia*, and *Nyssorhynchus* transmitting the malaria parasite to humans [2]. *Anopheles* species are often found in morphologically identical sibling species complexes, with approximately thirty species complexes identified in various parts of the world [3–6]. Each complex has a different number of sibling species, and 145 species have been identified [2]. The accuracy of morphological identification depends on the known ability to differentiate species and those held within species complexes cannot usually be differentiated morphologically. Cross-mating tests, mitotic and meiotic karyotypes, and molecular procedures are applied to identify sibling species within complexes [3–5]. Sibling species held within complexes often express different behaviours and bionomics, therefore, it is crucial to utilize molecular techniques to identify the mosquito specimens to species level, when examining the mosquito geography, ecology, and biology [3, 6].

More than eighty *Anopheles* species were identified in Indonesia, of which twenty-six are malaria vectors. So far only a few species have been studied as *Anopheles* species complex in Indonesia, including *Anopheles sundaicus*, *Anopheles maculatus*, *Anopheles barbirostris* and *Anopheles punctulatus* [7–13]. This does not rule out the possibility of other complexes that have not been discovered yet.

*Anopheles vagus* (sub-genus *Cellia*) was discovered in 1902 by Doenitz in Indonesia [14], and its subspecies, *An. vagus limosus* was initially reported in the Philippines [14, 15]. In Indonesia, *An. vagus* is a vector of malaria, filariasis, and Japanese encephalitis [16–18]. Except for Papua, practically all Indonesian Islands have *An. vagus* populations [19], which typically feed on cattle and other animals. These primary topographic zones comprise a diverse habitat, including brackish water, coastal plains, inland, hills, and mountains [20]. *Anopheles vagus* larvae are generally found in locations with calm or light water flow, such as puddles, on the beach, springs, the edges of rice fields, muddy ponds, animal tracks, and artificial containers such as old tires, drums, and on boats [20–22].

*Anopheles vagus* is predominantly a zoophilic, exophilic, and exophagic vector found in Asia [19]. However, in some previous studies, *An. vagus* was also reported to be slightly more anthropophilic, i.e. feeding on human blood and/or animal blood [23, 24]. This opportunistic behaviour can make these mosquitoes capable of transmitting *Plasmodium.* Therefore, these mosquitoes are now regarded as secondary malaria vectors, after the detection of *Plasmodium* spp. both by biological assays and molecular examination, which has been carried out in several regions in India, Bangladesh, Thailand, China, and especially Indonesia [19, 25–31].

This review evaluated the current evidence to determine whether *An. vagus* was a cryptic or sibling species, as well as the behavioural and genetic variation of *An. vagus* in Asia.

## Methods

### Literature search

This review was conducted according to the Preferred Reporting Items for Systematic Reviews and Meta-Analysis (PRISMA) 2020 guidelines [32]. The databases Pubmed, Scopus, Europe PMC, and Proquest were searched using the following keywords: “(Anopheles) AND (Anopheles vagus)”; “((species complex) OR (sibling species)) AND (Anopheles vagus)”. The search was conducted from February to November 2022.

### Eligibility criteria and study selection

English and Indonesian language full articles concerning *An. vagus*, various systematic examinations of species complex based on karyotypic identification, cross-mating experiments, morphometric and morphological investigations of palps and wings, and molecular investigations for identifying species and phylogenetic analysis were evaluated. Three authors (DL, DS, CA) independently extracted data regarding authorship, years, country, study populations, and key findings from each study.

## Results

**Fig. 1:**
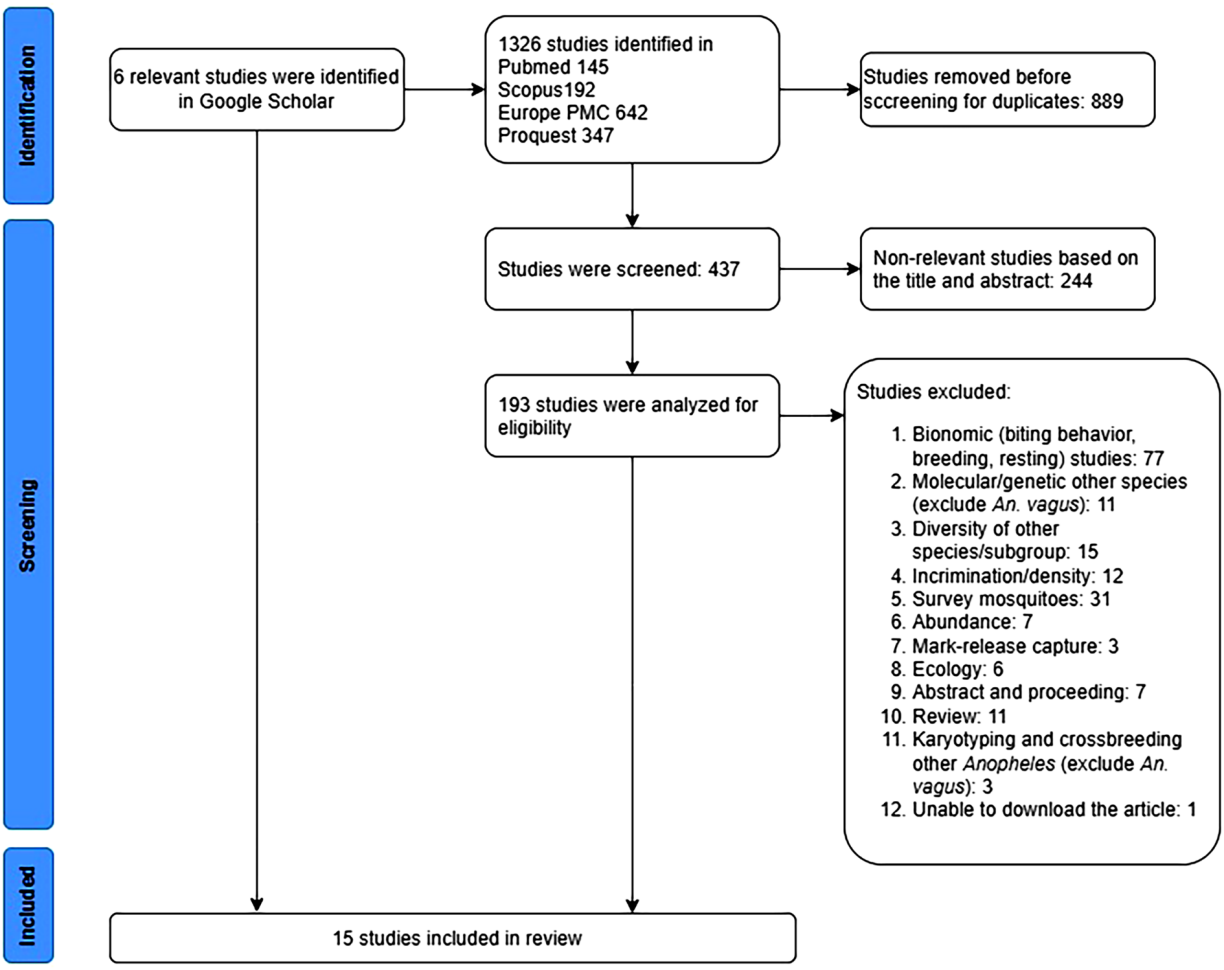
PRISMA flow diagram of literature searching

**Fig. 2:**
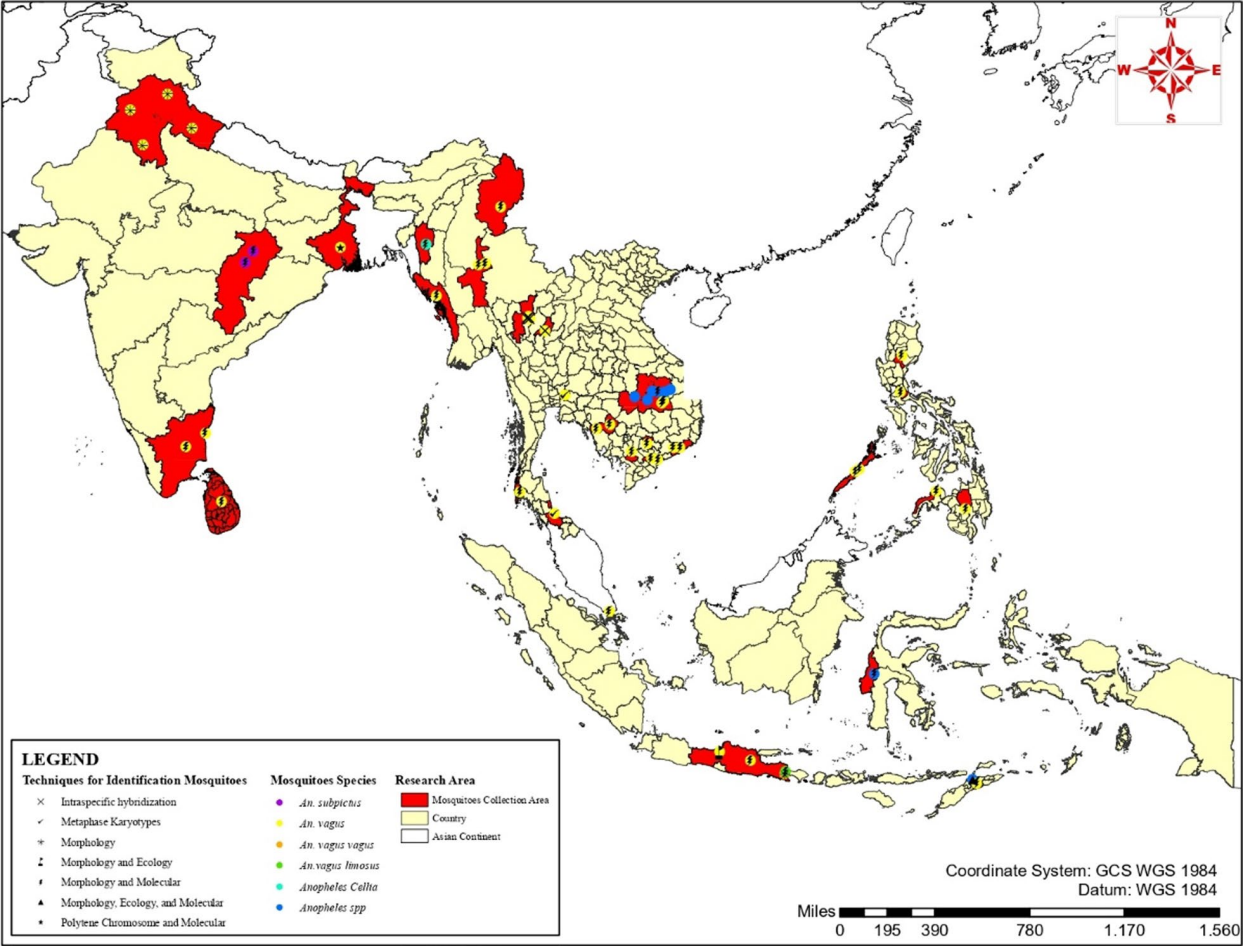
Geographic Distribution *Anopheles *spp. and *Anopheles vagus* examination. Map of Country in Mainland Asia and South East Asia was sourced from: https://gadm.org/maps.html

In total, 145 articles were identified in Pubmed, 192 in Scopus, 642 in Europe PMC, and 347 in Proquest, with a further six relevant studies in Google Scholar (Fig. 1). Most studies were about *Anopheles* spp*.*, focusing on bionomy, survey mosquitoes and their role as a vector, incrimination study, abundance and diversity, and molecular analysis. Only a few articles focused on *An. vagus* and the systematic study of the possibility of a species complex. Of the fifteen studies reviewed [33–47] (Table 1), seven studies examined a sample of *An. vagus* from the Indonesian archipelago (Central Java, East Java, and West Sulawesi Indonesia as well as Dili East Timor), four studies involved samples from India, two from Thailand, one study from the population in 18 areas of mainland Asia and Southeast Asia, and one study from the border regions of Laos and Cambodia (Fig. 2). Of the fifteen articles on *An. vagus*, all have used different species name in the articles and in the genebank, such as *An. vagus*. *An. limosus*, *An. vagus vagus* and *An. vagus limosus* [36, 37, 42].

**Table 1:** Summary and key findings included articles

Techniques for identification mosquito siblings	Authors/years	Population size/sample site	Key findings	References
Metaphase karyotypes	Baimai et al*.* 1996^a^	*Anopheles* subgenus *Cellia* from Thailand, Indonesia, Filipina, and Bangladesh, for *An. vagus* sample from Thailand (Songkhla and Nakhon Nayok province)	Two forms of isoline F1 *An. vagus* progeny: form A from southern Thailand Chiangmai and Songkhla; form B from Nakhon Nayok Province, Thailand	[33]
Intraspecific hybridization	Choocote et al*.* 2002^a^	*An. vagus* from San Sai and San Kamphaeng Districts, Chiang Mai Province, northern Thailand	All crosses yielded viable progeny, with no evidence of genetic incompatibility between *An. vagus* forms A and B from a different area (allopatric). The eggs of *An. vagus* forms A and B were morphometrically and morphologically identical	[34]
Polytene chromosome and molecular	Paul & Banerjee, 2016^b^	*An.vagus* from west Bengal, India	*An. vagus* from a different area (allopatric) in west Bengal has a different arm structure of the polytene chromosome, with the presence of tetramers, pentamers, polymers, and the absence of repeats in the ITS2 sequence	[40]
Morphology	Jagdish Kaur, 2015^b^	*An. vagus* and *An. fluviatilis f*rom Punjab, Haryana, Uttarakhand, and Himachal Pradesh, India	Thirteen morphological variations were observed in the ornamentation of the wings and palpi (7 variations in the wings and palpi of *An. vagus* and 6 wing variations in *An. fluviatilis* (both from allopatric and sympatric area)	[41]
Morphology	Wahyuni et al. 2018^b^	*An. vagus vagus* and *An. vagus limosus* from Banyuwangi East Java, Indonesia	*An. vagus* found in Bangsring Village does not have a combination variation in the proboscis and prehumeral wings. *An. vagus vagus* is characterized by unspotted legs, a proboscis with pale bands, and prehumeral wings with pale bands between two dark bands, whereas *An. vagus limosus* has characteristic unspotted legs, a dark overall proboscis, and prehumeral wings with dark bands; there is no mixed combination of these characteristics between the two species	[42]
Morphology and ecology	Siti Alfiah & Mujiyono, 2014^b^	*An.vagus* from Semarang, Central Java Indonesia	The variations were in the size and the number of hair branches and filaments. This variation in the intra and interpopulation *An. vagus* in fresh and brackish water was caused by the difference in geographical location (allopatric speciation)	[43]
Morphology, ecology, and molecular	Cooper et al. 2010^a^	*Anopheles spp* from Dili East Timor	Analysis of ITS2 and Cyt b revealed the *An. vagus* genotype A (mainly found inland and genetically similar to *An. subpictus*) and *An. vagus* genotype B (dominant in HLC and + sporozoite, mainly found on the coast and brackish water genetically similar to *An. sundaicus*)	[44]
Morphology and molecular	Zarowiecki et al. 2011^a^	*An. vagus* from 18 populations in 10 countries in mainland Asia and Southeast Asia	Analysis of COI (Cytochrome Oxidase I) and ITS2 (Internal Transcribed Spacer 2) revealed that *An. vagus* appears to reflect a highly diverse, monospecific, widespread taxon distributed in Sri Lanka and India throughout mainland and Southeast Asia, except Java and East Timor (*An. vagus* FJ654649), which forms a distinct genetic lineage with the sample from other regions. There is some degree of east–west genetic differentiation in *An. vagus* along the Thai-Myanmar border due to historical allopatric fragmentation	[45]
	Zomuanpuii et al. 2013^a^	*Anopheles* subgenus Cellia (10 species) from 5 districts in Mizoram, India	Analysis of ITS2: *An. vagus* had the longest ITS2 regions but possesses low repeats and polynucleotide microsatellites (no dimer repeats found in *An. vagus*)	[46]
	Paul et al. 2015^b^	*An. vagus* and *An. subpictus* from Morga India	3 different palp types from two seasons (summer and monsoon) in 7 samples; *An. vagus* is genetically similar to *An. subpictus*	[47]
	Davidson et al. 2020^a^	*Anopheles spp* from Karama, West Sulawesi Indonesia	Analysis of COI and ITS2 sequence: 2 distinct groups identified as *An. vagus* (AN4 and AN5) from this sympatric area, with *An. vagus* (AN4) more closely related to *An. sundaicus* (AN17) and *An. vagus* (AN5) more closely related to *An. subpictus*	[35]
	Senjarini et al. 2021^b^	*An. vagus vagus* and *An. vagus limosus* from Banyuwangi, East Java Indonesia	Analysis of ITS2 sequence: *An. vagus vagus* and *An. vagus limosus* closely related to *An. vagus* FJ654649 and were in the same clade	[36]
	Senjarini et al. 2021^a^	*Anopheles spp* from Banyuwangi, East Java Indonesia	Analysis of Sma-ITS 2: *An. vagus vagus*, *An.vagus limosus*, and *An. indefinitus* formed a single clade with no clear boundaries between *An. vagus vagus* and *An. vagus limosus*	[37]
	Zhang et al. 2022^a^	*Anopheles spp.* from the Cambodia-Laos border	*An. vagus* was the dominant species but only 12 *An.vagus* were randomly investigated by molecular analysis of ITS 2 and the COII sequence: all are not genetically distinct *An. vagus*-like species	[38]
	Hasanah et al*.* 2022^a^	*An. vagus, An. subpictus, An. sundaicus,* and *An. aconitus* from basring village Banyuwangi, East Java, Indonesia	Analysis of ITS2 sequence: *An. vagus* and *An. aconitus* were monophyletic and *An. subpictus* and *An. sundaicus* were polyphyletic	[39]

^a^Studies from the Pubmed, Scopus, Europe PMC and ProQuest databases

^b^Study from Google scholar

In this review, the members of *An. vagus *sensu lato (s.l.) grouped based on the morphology (*An. vagus*, *An. vagus vagus*, *An. vagus limosus*, and *An. limosus*), karyotyping (form A and B), and molecular (*An. vagus* genotype A and B) and *An. vagus* AN4 and AN5.

### Current grouping of *Anopheles vagus*

Female *An. vagus* was initially discovered by Doenitz (1902) in Sumatera, meanwhile the male *An. vagus* was discovered in Java and other regions in Indonesia [14]. Based on morphological features, *An. vagus* can be divided into three subspecies: *An. vagus vagus*, *An. vagus limosus* and *An. vagus albino* [48]. *Anopheles vagus limosus* was found in Luzon Island, Philipines, and *An. vagus vagus* was found in Lake Lanao, located in Lanao Plateau, Philippines [49]. The status of *An. vagus limosus* was later promoted as a new species by Ramalingan et al*.* [50], who collected the two subspecies at the sympatric speciation in Sabah, Malaysia. This separation was reinforced by developing the cladistic classification of the genus *Anopheles,* which placed *An. vagus* and *An. limosus* into different species but still siblings in Pyretophorus series based on morphological and morphometric characters [51–53].

### Morphology-based grouping of *Anopheles vagus*

One of the distinctive characteristics of the genus *Anopheles* is its morphological variety. *Anopheles vagus* differs from other species in the Pyretophorus series, such *An. indefinitus* and *An. subpictus* from its pale apical band on its proboscis. It also differs from *An. limosus* from it lacks spots on its femur and tibia. *Anopheles vagus* has three or more palpi with pale or dark bands, a pale band at the proboscis's tip, and a pale band between the two dark bands on the humeral wing [54]. The species of *An. vagus* and *An. limosus* are distinguished by the proboscis and prehumeral region variations, which may be a combination of traits from two species specimens from Java, Indonesia [45]

The current study from India found that the *An. vagus* palp variation is discernible from the uneven and black tip palp [47]. The size and number of the collected branches, hairs, and filaments may vary depending on whether the collected *An. vagus* specimens were from freshwater or brackish water or due to the season or geography [41, 43, 47]. However, in Bangsring, East Java, the sample population taken from an area near a brackish water pond showed that the two subspecies of *An. vagus* do not have a combination variation in the proboscis and prehumeral wings. *Anopheles vagus vagus* is characterized by unspotted legs, a proboscis with pale bands, and prehumeral wings with pale bands between two dark bands, while *An. vagus limosus* has characteristic unspotted legs, a dark overall proboscis, and prehumeral wings with dark bands [42].

### Karyotype grouping of *Anopheles vagus*

Several mosquitoes of the subgenus *Cellia*, including a few *An. vagus* mosquitoes from Thailand were used in the initial studies on metaphase analysis of karyotypes [33]. Two distinct *An. vagus* karyotypes, form A and form B, were identified with a different length of the chromosomal arm. However, it has yet to be determined if the two metaphase karyotypes represent intra- or interspecies variations [33].

Experimental intraspecies cross-mating was conducted to identify the post-mating barriers between isolines of *An. vagus* types A and B from the districts of San Sai and San Kamphaeng, Chiang Mai Province, Northern Thailand. The results revealed that the *An. vagus* eggs are homogeneous with both forms, proving that the *An. vagus* species has two cytological forms that are polymorphic races [34]. In addition to karyotyping, there is only one study that analyses polytene chromosomes in *An. vagus.* Therefore, the result must be different from other studies. In that study, it was revealed that distinct polytene chromosomal arms variation in banding and puffing patterns with tetramers, pentamers, and polymers present but no tandem repeats in the ITS2 sequences [40].

### Molecular grouping of *Anopheles vagus*

The advent of molecular technologies in the early 1990s has provided new insights into mosquito taxonomy and further strengthened the taxonomic classification mainly based on morphological features. Molecular barcoding using either the internal transcribed spacer 2 (ITS2) fragment of the nuclear ribosomal gene or the mitochondrial cytochrome oxidase I (COI), and NADH dehydrogenase subunit 6 (ND6) gene offered complementary evidence to the current taxonomic classification and identification of a cryptic species [55–58].

**Fig. 3:**
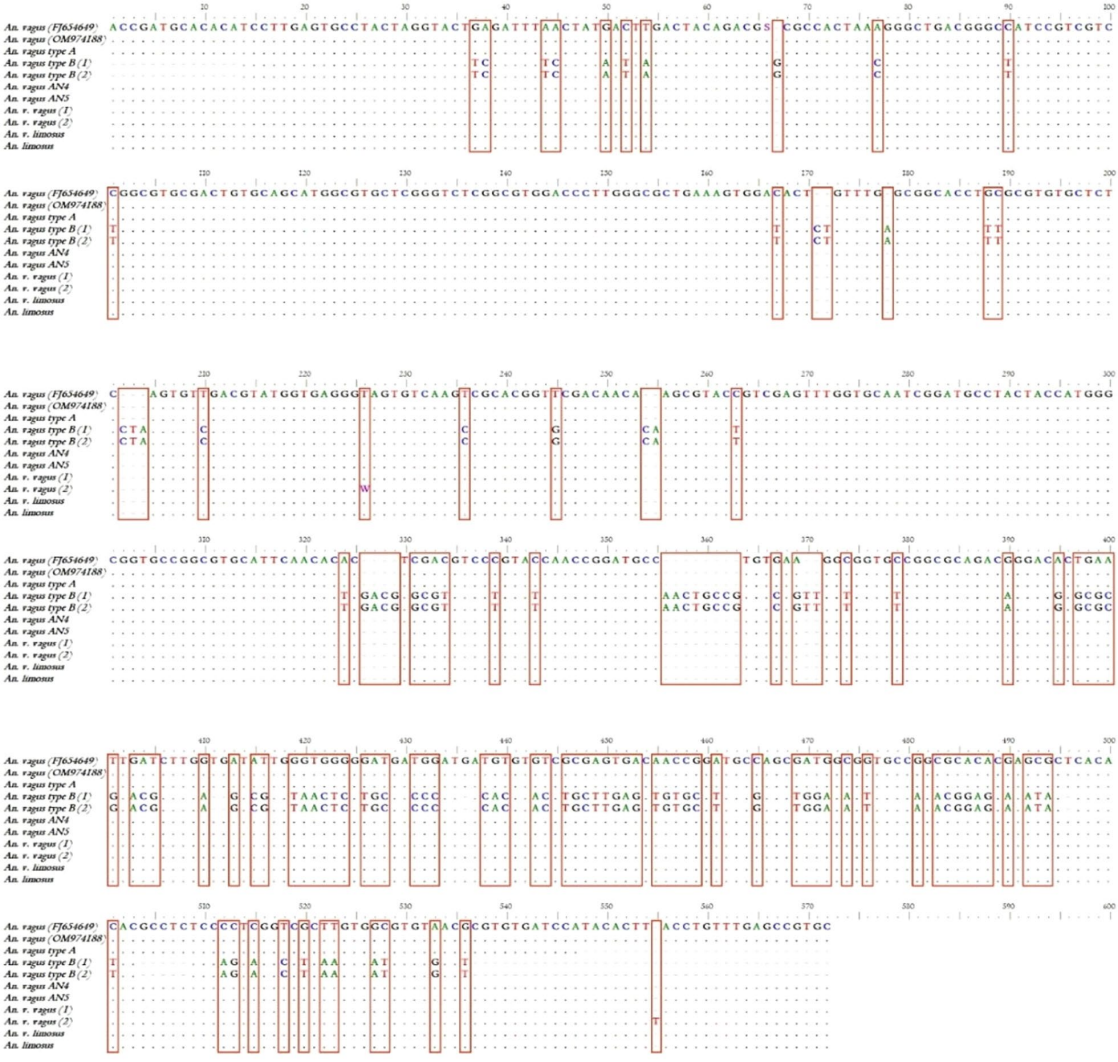
Sequences Alignment rDNA ITS2 fragment sample *An. vagus* members from Indonesia and East Timor. *An. vagus* (GenBank accession number FJ654649) is the reference sequence from all studies from Indonesia and East Timor. *An. vagus* (OM974188) is newest sequence sample from East Java Indonesia. GenBank accession number GQ500122 for *An. vagus* genotype A, GQ480824 and GQ480823 for *An. vagus* Genotype B, MT740902 and MT740903 for *An. vagus* AN4 dan AN5, MW314227 and OL437110 for *An. vagus vagus* (1) and (2), MW319822 for *An. vagus limosus*. OL437109 for *An. limosus*

**Fig. 4:**
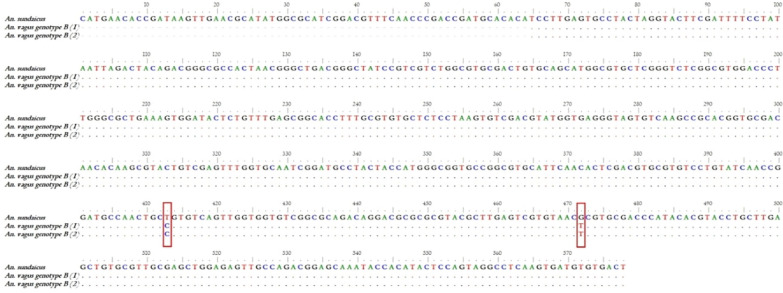
Sequence alignment rDNA ITS2 fragment *An. sundaicus* (AY768540) and *An. vagus* genotype B (GQ480823 and GQ480824)

*Anopheles vagus* appears dominant in mainland and Southeast Asia populations comprising a molecularly highly diverse yet monospecific, widely dispersed taxon. Interestingly, specimens from Java and East Timor in the Indonesia Archipelago region had a unique genetic lineage to other *An. vagus* in Asia [45]. *Anopheles vagus* had a highly conserved ITS2 region containing over 500 bp and flanked by 5.8S rDNA in the upstream and large rDNA in the downstream (Fig. 3). A recent study by *An. vagus* ITS2 revealed no genetic variations among the *An. vagus* populations from Bangsring, East Java, Indonesia [39]. Likewise, there were no genetically distinct *An. vagus*-like species in twelve specimens from the border between Laos and Cambodia [38]. In contrast, studies conducted in East Timor [44] reported two groups of *An. vagus* based on ITS2 sequence; Genotype A perfect match with sequence of *An. vagus* from East Timor and East Java (GenBank accession number FJ654649), whereas Genotype B was morphologically identical to *An. vagus*, but the ITS2 sequence was nearly identical to *An. sundaicus* (Fig. 4). The *An. vagus* genotype B was reportedly more anthropophilic than the *An. vagus* genotype A as it was more frequently collected through the HLC technique.

Interestingly, two groups of sympatrically distributed *An. vagus* was also reported in the remote inland village of Karama, West Sulawesi, Indonesia; one was closely related to *An. sundaicus*-like, namely AN4, while the other was related to *An. subpictus* like (AN5) [35]. However, no further information as to whether the two *An. vagus* subgroup was reproductively isolated [47]. Both these studies used the genetic marker internal transcribed spacer 2 (ITS2). These studies also used COI sequences to identify their phylogenetic relationship.

*Anopheles vagus vagus* and *An. vagus limosus* from Bangsring, East Java, Indonesia are morphologically different, but their phylogenetic relationship was very close with ITS2 sequence similarity of more than 99%. Both *An. vagus vagus* and *An. vagus limosus* ITS2 sequence resembles with the previously published *An. vagus* ITS2 sequence (GenBank accession number FJ654649) [20, 23].

## Discussion

The biological and ecological notion of allopatric speciation which enables the *Anopheles* species to be separated in an ecological niche, involves ecosystem patterns, geographic barriers, and varied terrain that result in physical separation and reproductive isolation [3, 6, 59, 60]. Initially, it was believed that it was unusual for gene flow to remain uninhabited; however, it is now widely acknowledged that sympatric speciation has occurred possibly involving interbreeding speciation and assortative mating by habitat or secondary gene flow [61–63].

*Anopheles vagus* currently possesses several sub-species based on morphological characteristics and karyotype. Karyotype forms A and B are distributed in allopatric and thus possess geographical barriers to mating but have been shown through laboratory cross-mating to produce viable offspring [35] thus, the finding they concluded that *An. vagus* form A and form B from allopatric speciation are likely sibling species rather than different species entities[34]

Morphological characteristics divide *An. vagus* into two subspecies; *An. vagus vagus* and *An. vagus limosus* [14, 15]. The latter subspecies was later promoted into a new species, *An. limosus* (based on Harbach classification for *Anopheles*) [2, 51–53]. *Anopheles vagus* and *An. limosus* were distributed sympatricly in several parts of Southeast Asia [42, 45, 50].

Analysis of ITS2 sequences of all members of *An. vagus *s.l. revealed that *An. vagus*, *An. vagus vagus*, *An. vagus limosus* and *An. limosus* share almost identical ITS2 sequences except for *An. vagus* genotype B is, in fact, a member of *An. sundaicus* [23]. Therefore, despite the distinct morphological variation, all members of *An. vagus* are possibly conspecific. Regarding *An. limosus* that has been given new species status, further species confirmation using the other barcoding marker such as COI and ND6 may support this new status. The other issue that is also important is whether the *An. vagus* and *An. limosus* naturally mate with each other. So far, despite their close relationship, there is no evidence that they could mate naturally or in a laboratory setting that produces viable offspring.

## Conclusion

Literature review studies revealed that *An. vagus* is possibly conspecific despite distinct morphological characteristics of *An. vagus* and *An. limosus.* Further information using mitochondrial COI and ND6 barcoding and experimental cross-mating between the *An. vagus* and *An. limosus* may provide additional evidence for the status of *An. vagus* as a species complex.

## Data Availability

All data are available.

## Abbreviations

ITS2: Internal transcribed spacer 2
COI: Cytochrome oxidase I
HLC: Human landing catch
ND6: NADH dehydrogenase subunit 6
